# Intravenous Immunoglobulin Induced Transaminitis

**DOI:** 10.7759/cureus.51347

**Published:** 2023-12-30

**Authors:** Aviraag Vijaya Prakash, Aparna Parvathaneni, Sarat Malempati, Gary Keilson

**Affiliations:** 1 Internal Medicine, Saint Vincent Hospital, Worcester, USA; 2 Internal Medicine, Gandhi Medical College, Hyderabad, IND; 3 Neurology, Saint Vincent Hospital, Worcester, USA

**Keywords:** intravenous immunoglobulin (ivig), elevated liver enzyme, ivig treatment, liver damage, small fiber neuropathy

## Abstract

Intravenous immunoglobulin (IVIG) is a common therapeutic modality used in immune-mediated neuropathy. While the therapeutic benefits are well known, adverse reactions have been reported. One such adverse event, though rare, is transaminitis, which appears to be a transient and a self-limiting adverse reaction. Though most of the cases implicate the stabilizing agent to be the culprit, the exact mechanism is unknown. Thus far, it has been speculated that maltose, which has been commonly used as a stabilizer, is the cause of IVIG transaminitis. We present a unique case of a patient who developed transaminitis post-IVIG in which glycine was used as a stabilizing agent. We aim to draw a potential association between IVIG therapy and the development of transaminitis, thereby providing insight into the underlying mechanisms, as well as clinical features, and possibly encouraging further research on this topic.

## Introduction

Small fiber neuropathy (SFN) is a ubiquitous problem complicating healthcare throughout the world and has been shown to affect more than 20 million people above the age of 40 years in the United States [1]. The etiology reported is the involvement of small myelinated Aδ and unmyelinated C nerve fibers from a myriad of triggers of metabolic, infectious, toxic, and autoimmune origin [2]. Recent studies have increasingly shown seropositivity with trisulfated heparin disaccharide (TS-HDS), fibroblast growth factor receptor 3 (FGFR-3), or plexin-D1 autoantibodies, and thus with the growing evidence of autoimmunity in SFN, IVIG has become a popular treatment choice [3]. Intravenous immunoglobulin (IVIG) is a polyclonal fraction obtained from a plasma pool of thousands of healthy donors and typically contains intact immunoglobulin G molecules with trace amounts of CD4, CD8, HLA molecules, and certain cytokines [4,5]. However, the use of IVIG is not devoid of complications. We describe the case of a patient who developed acute liver injury following the administration of IVIG.

## Case presentation

A 53-year-old female presented with complaints of nausea and vomiting, which had significantly limited her appetite for about a week. At presentation, she was recognized to have hyponatremia with a sodium of 121 and was admitted to the hospital for the management of acute hyponatremia. Her only significant past medical history was SFN with complaints of pain and burning sensation in her hands and feet from around 15 years.

Her diagnosis of SFN had been established about a year and a half before hospitalization. An extensive panel of laboratory tests that included a complete blood count (CBC), comprehensive metabolic panel, thyroid function tests, heavy metal screen, and autoimmune panel apart from positive anti-TS-HDS was unremarkable. Finally, a skin biopsy was obtained, which was diagnostic of SFN. She was hence started on IVIG after a failed trial of duloxetine, gabapentin, and pregabalin. After starting therapy, she noted improvement in her symptoms of pain and paresthesia. The IVIG she had been receiving was an FDA-approved, glycine-based immune globulin infusion called GAMMAGARD LIQUID, which has been associated with hypersensitivity, renal dysfunction, thrombotic events, aseptic meningitis, hemolysis, transfusion-associated lung injury, and transmittable infectious agents such as viruses. She was on a cycle of five days per week every four weeks, which was tapered gradually, and had received her last dose one week before the hospitalization.

On a routine physical, her vitals were noted to be stable, and the rest of her examination was grossly unremarkable. Initial laboratory investigations included a CBC and metabolic panel. The CBC was grossly normal apart from mild leukopenia at 3.6 cells/L with a normal differential. The metabolic panel was essentially normal apart from a deranged hepatic panel (Table 1). The erythrocyte sedimentation rate was elevated to 81 mm/hour with a normal C-reactive protein at 4.25 mg/L. A complete infectious disease work-up including tests for hepatitis was found to be non-contributory. She did have sporadic episodes of diarrhea, which, coupled with the finding of elevated transaminases, warranted a celiac disease evaluation, which was found to be negative. A thyroid panel was also found to be normal. Her R factor was 6, indicative of a hepatocellular pattern of injury. She reported no use of new medications, supplements, or increased alcohol intake. There was no rationale for diagnostic imaging, and thus none were obtained. After exhausting all relevant diagnostic tests and given the history of recent IVIG infusions, the etiology of her hepatic injury was thought to be from the IVIG administrations. She was managed supportively during her hospital stay with IV fluids, and her symptoms resolved. She was discharged home with a plan for prompt cessation of further IVIG administrations. A repeat hepatic functional panel around three weeks following discharge showed a drastic reduction of her liver enzymes (Table 1).

**Table 1:** Trend of the liver enzymes AST, aspartate transaminase; ALT, alanine transaminase

	Day 1	Day 2	Day 3	Day 21
AST (normal: 0-40 U/L)	541	460	395	84
ALT (normal: 0-32 U/L)	354	330	335	67
Alkaline phosphatase (normal: 25-150 U/L)	176	157	157	110

## Discussion

The immunomodulatory roles of IVIG are multifold and include blocking the Fc receptors of immune cells in the reticuloendothelial system, abrogation of pathogenic autoantibodies by idiotype-anti-idiotypic networks, and preventing complement cellular damage by exhaustion of C5b-9 membrane attack complex by clearing active complement products (C3b and C4b) [6]. IVIG, at its inception, had an increasing association with adverse effects such as fever, chills, fatigue, and joint pains, and this was thought to be due to the formation of immunoglobulin aggregates, and thus stabilizers such as sucrose, maltose, sorbitol, and glycine were added. They were also shown to contribute to modulating mononuclear cell proliferation and thus have since become a common additive in the preparation of commercial IVIG [7].

A rare side effect that has been previously described in connection with the use of IVIG is acute liver injury [8-10]. The mechanism of this pattern of injury has not been understood to date. One study involving 341 infusions of IVIG hypothesized that the use of saccharide stabilizing agents such as maltose and sucrose contributed to transaminitis [11]. The same study found that there were no elevations in liver enzymes among patients who were given glycine-based IVIG. Our case, however, is asynchronous with this analysis as on investigation, we found that the stabilizing agent in the IVIG formulation used in our patient was glycine, with no portion of saccharides, sorbitol, or glycol.

A previously proposed mechanism of IVIG-related cell injury was the induction of programmed cell death by involving Fas (CD95/APO-1), a transmembrane glycoprotein acting as an archetypal death receptor and activation of caspases [12]. This study, however, was specific to apoptosis induced by IVIG in CD-40-activated normal tonsillar B cells and leukemic cells of lymphocyte and monocytic lineage. There is still a dilemma as to which components of IVIG are involved in cell damage, warranting a further look into the individual components of IVIG.

## Conclusions

With the failure to completely understand the etiopathogenesis involved in this specific disease pattern, one consistent positive observation is the transient nature of the liver injury. With the cessation of IVIG, there has been a demonstration of resolution of transaminitis both in our patient and in previous studies. Thus far, we have not come across a fatal or disabling case of liver injury from the use of IVIG, and thus further exploration into the pathogenesis by invasive methods such as liver biopsy is needed.
